# Crystal structure of dimethyl 2-((2*Z*,5*Z*)-5-(2-meth­oxy-2-oxo­ethyl­idene)-2-{(*E*)-[2-methyl-5-(prop-1-en-2-yl)cyclo­hex-2-enyl­idene]hydrazinyl­idene}-4-oxo­thia­zolidin-3-yl)fumarate

**DOI:** 10.1107/S2056989017001190

**Published:** 2017-01-31

**Authors:** Abdellah N’ait Ousidi, My Youssef Ait Itto, Aziz Auhmani, Abdelkhalek Riahi, Abdelwahed Auhmani, Jean-Claude Daran

**Affiliations:** a Laboratoire de Physico-Chimie Moléculaire et Synthèse Organique, Département de Chimie, Faculté des Sciences, Semlalia BP 2390, Marrakech 40001, Morocco; b Institut de Chimie Moléculaire de Reims, CNRS UMR 7312 Bât., Europol’Agro - Moulin de la Housse UFR Sciences BP 1039-51687 Reims Cédex 2, France; c Laboratoire de Chimie de Coordination, CNRS UPR8241, 205 route de Narbonne, 31077 Toulouse Cedex 04, France

**Keywords:** crystal structure, heterocyclic compounds, thia­zolidine derivatives, natural product

## Abstract

The crystal structure and the conformation of the title compound, C_22_H_27_N_3_O_7_S, were determined from the synthetic pathway and by X-ray analysis. This compound is a new 4-thia­zolidinone derivative prepared and isolated as pure product from thio­semicarbazone carvone. The mol­ecule is built up from an oxo­thia­zolidine ring tetra­substituted by a meth­oxy–oxo­ethyl­idene, a maleate, an oxygen and a cyclo­hexyl­idene–hydrazone. The cyclo­hexyl­idene ring is statistically disordered over two positions, resulting in an inversion of configuration for the substituted carbon.

## Abstract 2

The crystal structure of the title compound, dimethyl 2-((2*Z*,5*Z*)-5-(2-meth­oxy-2-oxo­ethyl­idene)-2-{(*E*)-[2-methyl-5-(prop-1-en-2-yl)cyclo­hex-2-enyl­idene]hydrazinyl­idene}-4-oxo­thia­zolidin-3-yl)fumarate displays a conformational disorder which inverts the configuration of the chiral C atom within the cyclo­hexyl­idene ring.

## Chemical context   

In recent years, the synthesis of heterocyclic systems containing nitro­gen and sulfur has attracted great inter­est because of their broad spectrum of pharmacological activities. The thia­zol nucleus is found in a large number of natural products (Nielsen *et al.*, 2012 ▸), as well as in diverse pharmaceutical products (Le Flohic *et al.*, 2005 ▸). Indeed, some 4-aryl­thia­zole derivatives exhibit a strong anti-inflammatory activity (Hirai & Sugimoto, 1977 ▸) while some tetra­hydro­thia­zolo-[4,5-*b*] pyridines show anti­oxidant properties (Uchikawa *et al.*, 1996 ▸). The therapeutic usefulness of these heterocyclic systems prompted us to prepare a new substituted thia­zole which shows important medicinal properties. The title compound **2** was synthesized by the reaction of (*R*)-thio­semicarbazone carvone **1** easily obtained from naturally occurring (*R*)-carvone] with dimethyl acetyl­enedi­carboxyl­ate in basic medium, using ethanol as solvent. The resulting product **2** [Chem scheme1] was obtained in 65% yield.
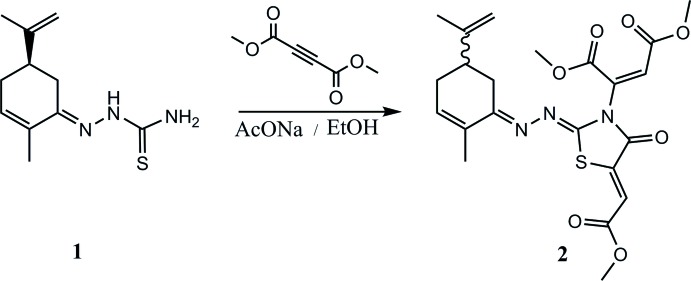


The structure of **2** [Chem scheme1] was established using spectroscopic (MS and NMR) data, while its stereochemistry was determined based mainly on the synthetic pathway and implied by the X-ray analysis. The thia­zolic compound **2** is finally identified as dimethyl 2-((2*Z*,5*Z*)-5-(2-meth­oxy-2-oxo­ethyl­idene)-2-{(*E*)-[2-methyl-5-(prop-1-en-2-yl)cyclo­hex-2-enyl­idene]hydrazinyl­idene}-4-oxo­thia­zolidin-3-yl)fumarate.

## Structural commentary   

**Figure 1:**
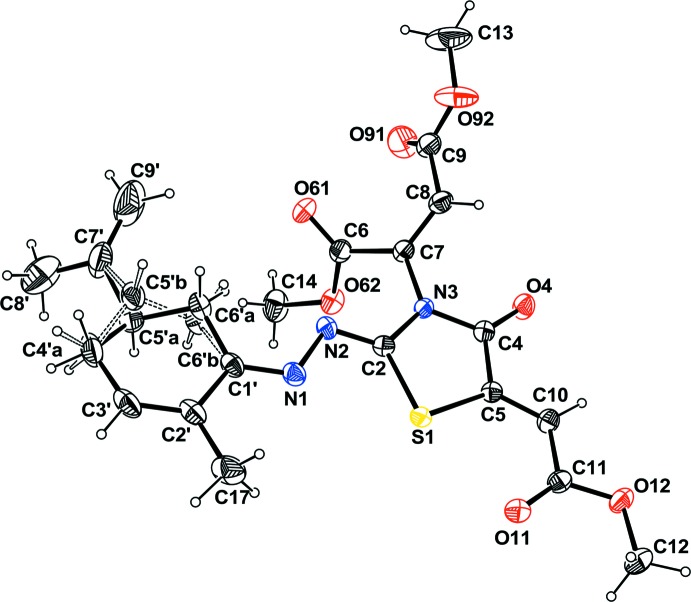
The mol­ecular view of the title compound with the atom-labelling scheme. Displacement ellipsoids are drawn at the 30% probability level. H atoms are represented as small circle of arbitrary radii. The disordered part is shown with dashed lines.

The title mol­ecule is built up from an oxo­thia­zolidine ring tetra­substituted by a meth­oxy-oxo­ethyl­idene, a fumarate, an oxygen and a cyclo­hexyl­idene-hydrazone (Fig. 1 ▸). As expected, the thia­zolidine ring and all the atoms attached to it (plane *A* = S1/C2/N3/C4/C5/N2/C7/O4/C10) are roughly coplanar with the largest deviation from the mean plane being 0.085 (2) Å for C10. The butadiene fragment (C1′/C2′/C3′/C4′*A/*C4′*B*) of the cyclo­hexyl­idene ring is twisted slightly with respect to this plane, making a dihedral angle of 8.3 (2)°. The meth­oxy­carbonyl group (C11/O11/O12/C12) is also twisted slightly with respect to plane *A*, with a dihedral angle of 8.2 (2)°. The meth­oxy­carbonyl groups (C6/O61/O62/C14 and C9/O91/O92/C13) of the fumarate group make dihedral angles of 70.06 (7) and 75.59 (9)°, respectively, with the thia­zolidine ring.

The most striking feature of this structure is the conformational statistical disorder which affects the cyclo­hexyl­idene ring: atoms C6′ and C5′ are split over two positions, each of half occupancy, with respect to the mean plane of the butadiene (C1′–C4′) fragment (Fig.1). Such disorder inverts the configuration at C5 (***R*** C5′*A* and ***S*** C5′*B*) and so the crystal might be considered as a racemate. Could the crystal be considered as a co-crystal built up from the combination of ***R*** and ***S*** configurations? It is difficult to answer this question.

## Supra­molecular features   

**Figure 2:**
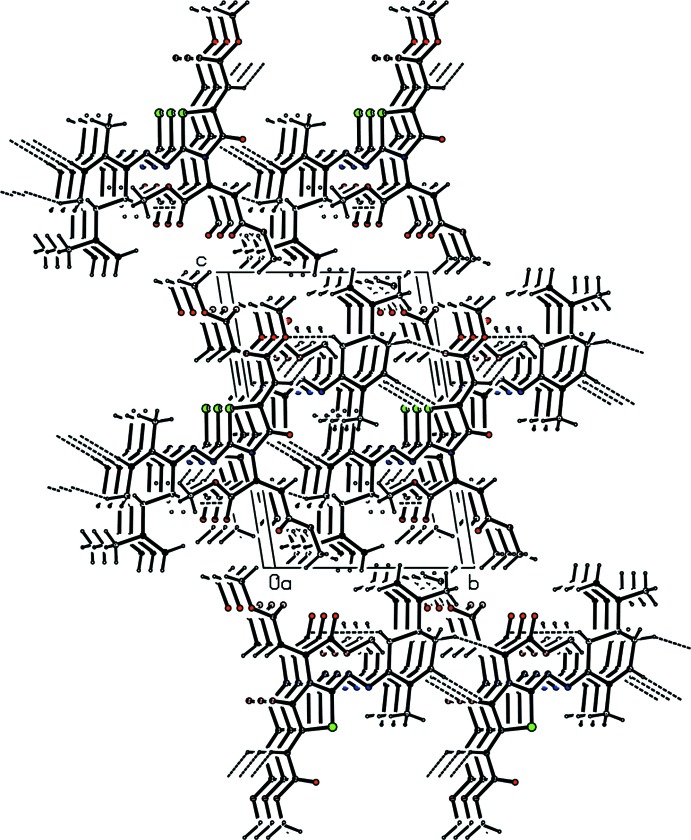
A packing view showing the formation of layers parallel to the (001) plane.

In the crystal, there are C—H⋯O weak hydrogen-bonding inter­actions (Table 1 ▸) which link the mol­ecules, building a two-dimensional network parallel to the (001) plane, as shown in Fig. 2 ▸.

## Database survey   

A search of the Cambridge Structural Database (CSD, Version 5.37, update November 2015; Groom *et al.*, 2016 ▸) using a thia­zolidine ring substituted by a hydrazone linked to a cyclo­hexyl ring as the main skeleton, revealed the presence of six structures.
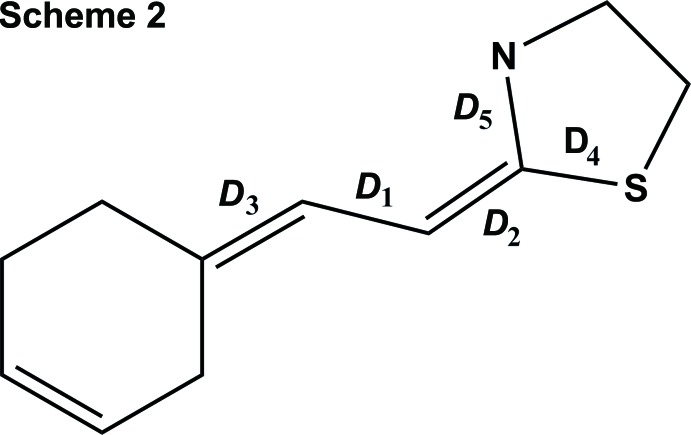


A comparison of the main C—N, N—N, C—S distances in the title compound and the structures extracted from the CSD shows good correlation: within the C=N—N=C fragment, the double bonds are located on the CN, the N—N distance is that of a single bond corresponding to a hydrazono group. The C=N—N=C torsion angles (Table 2 ▸) indicate that in each case the four atoms are nearly planar.

## Synthesis and crystallization   

A solution of (1*R*)-thio­semicarbazone carvone **1** and dimethyl acetyl­enedi­carboxyl­ate (1.25 eq) in anhydrous MeCN (50 mL), was heated under reflux for 30 min. After the completion of the reaction (the progress of the reaction was monitored by TLC), the solvent was evaporated to dryness. The crude product was purified by silica gel chromatography (230–400 mesh) using hexa­ne/ethyl acetate (95:5) as eluent. The pure thia­zolic product **2** was obtained in 65% yield. Slow evaporation from an ethano­lic solution of the title compound gave crystals of **2** suitable for crystallographic analysis.

## Refinement   

Crystal data, data collection and structure refinement details are summarized in Table 3 ▸. The disorder was been refined using the tools available in *SHELXL2014*. All H atoms were initially located in a difference Fourier map but were placed in geometrically idealized positions and constrained to ride on their parent atoms with C—H = 0.95–1.0 Å and O—H = 0.84 Å, with *U*_iso_(H) = 1.5*U*_eq_(C) for methyl H atoms and 1.2*U*_eq_(C,O) for all other H atoms.

## Supplementary Material

Crystal structure: contains datablock(s) I, global. DOI: 10.1107/S2056989017001190/xu5897sup1.cif

Structure factors: contains datablock(s) I. DOI: 10.1107/S2056989017001190/xu5897Isup2.hkl

CCDC reference: 1529291

Additional supporting information:  crystallographic information; 3D view; checkCIF report

## Figures and Tables

**Table 1:** Hydrogen-bond geometry (Å, °)

*D*—H⋯*A*	*D*—H	H⋯*A*	*D*⋯*A*	*D*—H⋯*A*
C6′*A*—H6′2⋯O12^i^	0.99	2.56	3.349 (8)	136
C3′—H3′⋯O4^ii^	0.95	2.57	3.510 (3)	170
C4′*B*—H4′4⋯O11^iii^	0.99	2.45	3.414 (4)	164
C10—H10⋯O62^iv^	0.95	2.47	3.244 (3)	138

Symmetry codes: (i) 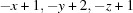; (ii) 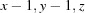; (iii) 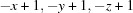; (iv) 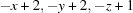.

**Table 2:** Comparison of main bond lengths and C=N—N=C torsion angles (Å, °) in the title compound and related structures For a definition of the distances *D*, see Scheme 2.

Refcode	*D*1	*D*2	*D*3	*D*4	*D*5	Torsion	
MUDRIO	1.406	1.277	1.287	1.769	1.386	179.0	
FOTQEM	1.417	1.269	1.292	1.756	1.380	173.8	
MIZJUC	1.407	1.281	1.291	1.761	1.392	179.4	
ROMXUN	1.414	1.278	1.278	1.749	1.367	−177.3	
WISTAV	1.429	1.256	1.278	1.753	1.413	−177.6	
WISTAV	1.412	1.290	1.288	1.758	1.354	177.2	
WURVAI	1.410	1.279	1.279	1.768	1.364	174.9	
This study	1.405 (3)	1.274 (3)	1.286 (4)	1.756 (3)	1.398 (3)	−168.9 (2)	

Reference: MUDRIO: Mohamed *et al.* (2015 ▸); FOTQEM: Gautam & Chaudhary (2015 ▸); MIZJUC: Mague *et al.* (2014 ▸); ROMXUN: Ramachandran *et al.* (2009 ▸); WISTAV: Gupta & Chaudhary (2013 ▸); WURVAI: Gautam *et al.* (2013 ▸).

**Table 3:** Experimental details

Crystal data	
Chemical formula	C_22_H_25_N_3_O_7_S
*M*_r_	475.51
Crystal system, space group	Triclinic, *P* 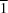
Temperature (K)	173
*a*, *b*, *c* (Å)	8.2468 (3), 9.8783 (4), 15.1039 (6)
α, β, γ (°)	96.144 (2), 105.172 (2), 95.750 (2)
*V* (Å^3^)	1170.14 (8)
*Z*	2
Radiation type	Mo *K*α
μ (mm^−1^)	0.19
Crystal size (mm)	0.37 × 0.25 × 0.03

Data collection	
Diffractometer	Bruker APEXII CCD
Absorption correction	Multi-scan (*SADABS*; Sheldrick, 2008 ▸)
*T*_min_, *T*_max_	0.732, 1.0
No. of measured, independent and observed [*I* > 2σ(*I*)] reflections	34166, 4778, 4085
*R*_int_	0.041

Refinement	
*R*[*F* ^2^ > 2σ(*F* ^2^)], *wR*(*F* ^2^), *S*	0.055, 0.123, 1.22
No. of reflections	4778
No. of parameters	315
No. of restraints	3
H-atom treatment	H-atom parameters constrained
Δρ_max_, Δρ_min_ (e Å^−3^)	0.30, −0.26

Computer programs: *APEX2* and *SAINT* (Bruker, 2014 ▸), *SHELXT2013* (Sheldrick, 2015*a* ▸), *SHELXL2014* (Sheldrick, 2015*b* ▸), *ORTEPIII* (Burnett & Johnson, 1996 ▸), *ORTEP-3 for Windows* (Farrugia, 2012 ▸), *Mercury* (Macrae *et al.*, 2008 ▸) and *PLATON* (Spek, 2009 ▸).

## supplementary crystallographic information

### Crystal data

**Table:**

C_22_H_25_N_3_O_7_S	*Z* = 2
*M**_r_* = 475.51	*F*(000) = 500
Triclinic, *P*1	*D*_x_ = 1.350 Mg m^−^^3^
*a* = 8.2468 (3) Å	Mo *K*α radiation, λ = 0.71073 Å
*b* = 9.8783 (4) Å	Cell parameters from 8618 reflections
*c* = 15.1039 (6) Å	θ = 2.4–26.8°
α = 96.144 (2)°	µ = 0.19 mm^−^^1^
β = 105.172 (2)°	*T* = 173 K
γ = 95.750 (2)°	Flattened, yellow
*V* = 1170.14 (8) Å^3^	0.37 × 0.25 × 0.03 mm

### Data collection

**Table:**

Bruker APEXII CCD diffractometer	4778 independent reflections
Radiation source: fine-focus sealed tube	4085 reflections with *I* > 2σ(*I*)
Graphite monochromator	*R*_int_ = 0.041
φ and ω scans	θ_max_ = 26.4°, θ_min_ = 2.6°
Absorption correction: multi-scan (SADABS; Sheldrick, 2008)	*h* = −10→10
*T*_min_ = 0.732, *T*_max_ = 1.0	*k* = −12→12
34166 measured reflections	*l* = −18→18

### Refinement

**Table:**

Refinement on *F*^2^	3 restraints
Least-squares matrix: full	Hydrogen site location: inferred from neighbouring sites
*R*[*F*^2^ > 2σ(*F*^2^)] = 0.055	H-atom parameters constrained
*wR*(*F*^2^) = 0.123	*w* = 1/[σ^2^(*F*_o_^2^) + (0.0088*P*)^2^ + 2.0702*P*] where *P* = (*F*_o_^2^ + 2*F*_c_^2^)/3
*S* = 1.22	(Δ/σ)_max_ < 0.001
4778 reflections	Δρ_max_ = 0.30 e Å^−^^3^
315 parameters	Δρ_min_ = −0.26 e Å^−^^3^

### Special details

**Table:**

Geometry. All esds (except the esd in the dihedral angle between two l.s. planes) are estimated using the full covariance matrix. The cell esds are taken into account individually in the estimation of esds in distances, angles and torsion angles; correlations between esds in cell parameters are only used when they are defined by crystal symmetry. An approximate (isotropic) treatment of cell esds is used for estimating esds involving l.s. planes.

### Fractional atomic coordinates and isotropic or equivalent isotropic displacement parameters (Å^2^)

**Table:**

	*x*	*y*	*z*	*U*_iso_*/*U*_eq_	Occ. (<1)
S1	0.62041 (8)	0.86199 (7)	0.53866 (5)	0.02031 (15)	
N1	0.3971 (3)	0.6620 (2)	0.40411 (16)	0.0238 (5)	
N2	0.4884 (3)	0.7458 (2)	0.35923 (16)	0.0219 (5)	
N3	0.6949 (3)	0.9353 (2)	0.38976 (15)	0.0197 (5)	
O4	0.8958 (2)	1.1256 (2)	0.45018 (13)	0.0279 (5)	
O11	0.7109 (3)	0.9837 (2)	0.72559 (14)	0.0310 (5)	
O12	0.8836 (3)	1.1828 (2)	0.78060 (13)	0.0298 (5)	
O61	0.5937 (3)	0.7601 (2)	0.16332 (14)	0.0376 (5)	
O62	0.7897 (2)	0.7213 (2)	0.28911 (14)	0.0282 (4)	
O91	0.7901 (4)	1.0015 (3)	0.12561 (17)	0.0515 (7)	
O92	0.6342 (3)	1.1721 (3)	0.13646 (17)	0.0538 (7)	
C2	0.5920 (3)	0.8376 (3)	0.41840 (18)	0.0197 (5)	
C4	0.7978 (3)	1.0332 (3)	0.46061 (18)	0.0199 (5)	
C5	0.7660 (3)	1.0086 (3)	0.55003 (18)	0.0191 (5)	
C1'	0.3108 (3)	0.5531 (3)	0.3521 (2)	0.0234 (6)	
C6'A	0.2869 (12)	0.5260 (9)	0.2500 (10)	0.0287 (17)	0.5
H6'1	0.3901	0.5660	0.2354	0.034*	0.5
H6'2	0.1910	0.5715	0.2179	0.034*	0.5
C5'A	0.2517 (8)	0.3731 (6)	0.2149 (4)	0.0268 (13)	0.5
H5'A	0.3538	0.3305	0.2444	0.032*	0.5
C4'A	0.1024 (4)	0.3074 (3)	0.2435 (3)	0.0405 (8)	0.5
H4'1	−0.0037	0.3307	0.2033	0.049*	0.5
H4'2	0.0986	0.2063	0.2335	0.049*	0.5
C6'B	0.3359 (11)	0.4961 (9)	0.2590 (10)	0.0287 (17)	0.5
H6'3	0.3707	0.5733	0.2283	0.034*	0.5
H6'4	0.4280	0.4377	0.2698	0.034*	0.5
C5'B	0.1756 (9)	0.4123 (7)	0.1957 (5)	0.0335 (15)	0.5
H5'B	0.0900	0.4759	0.1758	0.040*	0.5
C4'B	0.1024 (4)	0.3074 (3)	0.2435 (3)	0.0405 (8)	0.5
H4'3	−0.0178	0.2782	0.2087	0.049*	0.5
H4'4	0.1626	0.2259	0.2407	0.049*	0.5
C3'	0.1105 (4)	0.3520 (3)	0.3419 (2)	0.0345 (7)	
H3'	0.0414	0.2975	0.3696	0.041*	
C2'	0.2076 (3)	0.4630 (3)	0.3939 (2)	0.0268 (6)	
C17	0.2189 (4)	0.5009 (4)	0.4944 (2)	0.0407 (8)	
H17A	0.1542	0.4280	0.5151	0.061*	
H17B	0.1721	0.5872	0.5028	0.061*	
H17C	0.3378	0.5124	0.5310	0.061*	
C6	0.6828 (3)	0.7956 (3)	0.24011 (19)	0.0250 (6)	
C7	0.6900 (3)	0.9341 (3)	0.29482 (18)	0.0212 (5)	
C8	0.6931 (3)	1.0514 (3)	0.26006 (19)	0.0265 (6)	
H8	0.6827	1.1316	0.2978	0.032*	
C9	0.7115 (4)	1.0664 (3)	0.1666 (2)	0.0322 (7)	
C10	0.8410 (3)	1.0963 (3)	0.62646 (18)	0.0228 (6)	
H10	0.9203	1.1714	0.6238	0.027*	
C11	0.8037 (3)	1.0792 (3)	0.71487 (19)	0.0237 (6)	
C12	0.8491 (5)	1.1760 (3)	0.8692 (2)	0.0388 (8)	
H12A	0.7274	1.1754	0.8618	0.058*	
H12B	0.9120	1.2561	0.9131	0.058*	
H12C	0.8846	1.0918	0.8927	0.058*	
C13	0.6425 (6)	1.2005 (5)	0.0456 (3)	0.0715 (14)	
H13A	0.7613	1.2236	0.0463	0.107*	
H13B	0.5807	1.2779	0.0291	0.107*	
H13C	0.5913	1.1191	0.0000	0.107*	
C14	0.7916 (4)	0.5857 (3)	0.2438 (3)	0.0421 (8)	
H14A	0.6766	0.5354	0.2253	0.063*	
H14B	0.8672	0.5364	0.2866	0.063*	
H14C	0.8322	0.5930	0.1889	0.063*	
C7'	0.2228 (5)	0.3479 (4)	0.1088 (3)	0.0488 (9)	
C9'	0.2088 (5)	0.2048 (5)	0.0776 (3)	0.0663 (12)	
H9'1	0.2373	0.1919	0.0185	0.100*	
H9'2	0.0924	0.1619	0.0693	0.100*	
H9'3	0.2871	0.1621	0.1237	0.100*	
C8'	0.2471 (7)	0.4446 (5)	0.0520 (3)	0.0794 (16)	
H8'1	0.2547	0.4159	−0.0087	0.095*	
H8'2	0.2562	0.5397	0.0737	0.095*	

### Atomic displacement parameters (Å^2^)

**Table:**

	*U*^11^	*U*^22^	*U*^33^	*U*^12^	*U*^13^	*U*^23^
S1	0.0217 (3)	0.0199 (3)	0.0195 (3)	0.0015 (2)	0.0057 (3)	0.0047 (2)
N1	0.0222 (12)	0.0232 (12)	0.0266 (12)	−0.0003 (9)	0.0081 (10)	0.0056 (10)
N2	0.0214 (11)	0.0193 (11)	0.0244 (12)	−0.0011 (9)	0.0067 (9)	0.0033 (9)
N3	0.0215 (11)	0.0198 (11)	0.0166 (11)	−0.0013 (9)	0.0049 (9)	0.0016 (9)
O4	0.0274 (11)	0.0287 (11)	0.0256 (11)	−0.0073 (8)	0.0094 (8)	−0.0002 (8)
O11	0.0389 (12)	0.0293 (11)	0.0240 (11)	−0.0007 (9)	0.0086 (9)	0.0054 (9)
O12	0.0362 (12)	0.0320 (11)	0.0185 (10)	0.0004 (9)	0.0066 (8)	−0.0025 (8)
O61	0.0493 (14)	0.0339 (12)	0.0207 (11)	−0.0022 (10)	−0.0007 (10)	−0.0019 (9)
O62	0.0235 (10)	0.0307 (11)	0.0292 (11)	0.0061 (8)	0.0064 (8)	−0.0015 (9)
O91	0.0797 (19)	0.0446 (15)	0.0402 (14)	0.0044 (13)	0.0363 (14)	0.0033 (11)
O92	0.0513 (15)	0.082 (2)	0.0399 (14)	0.0191 (14)	0.0174 (12)	0.0396 (14)
C2	0.0187 (13)	0.0215 (13)	0.0199 (13)	0.0032 (10)	0.0064 (10)	0.0040 (11)
C4	0.0173 (12)	0.0207 (13)	0.0214 (13)	0.0040 (10)	0.0048 (10)	0.0021 (11)
C5	0.0165 (12)	0.0206 (13)	0.0201 (13)	0.0030 (10)	0.0044 (10)	0.0032 (10)
C1'	0.0186 (13)	0.0208 (13)	0.0320 (15)	0.0024 (10)	0.0072 (11)	0.0080 (12)
C6'A	0.032 (5)	0.020 (4)	0.035 (3)	−0.001 (3)	0.012 (4)	0.003 (3)
C5'A	0.023 (3)	0.027 (3)	0.028 (3)	0.005 (3)	0.001 (3)	0.005 (3)
C4'A	0.0361 (18)	0.0239 (16)	0.054 (2)	−0.0081 (13)	0.0055 (16)	0.0035 (15)
C6'B	0.032 (5)	0.020 (4)	0.035 (3)	−0.001 (3)	0.012 (4)	0.003 (3)
C5'B	0.026 (4)	0.026 (3)	0.042 (4)	−0.001 (3)	−0.003 (3)	0.006 (3)
C4'B	0.0361 (18)	0.0239 (16)	0.054 (2)	−0.0081 (13)	0.0055 (16)	0.0035 (15)
C3'	0.0262 (15)	0.0279 (16)	0.050 (2)	−0.0018 (12)	0.0095 (14)	0.0179 (14)
C2'	0.0201 (13)	0.0237 (14)	0.0392 (17)	0.0035 (11)	0.0089 (12)	0.0135 (12)
C17	0.0356 (18)	0.049 (2)	0.0427 (19)	−0.0017 (15)	0.0182 (15)	0.0174 (16)
C6	0.0251 (14)	0.0271 (15)	0.0225 (14)	−0.0019 (11)	0.0086 (11)	0.0020 (11)
C7	0.0192 (13)	0.0246 (14)	0.0192 (13)	−0.0010 (10)	0.0062 (10)	0.0015 (11)
C8	0.0262 (14)	0.0322 (16)	0.0200 (14)	−0.0001 (12)	0.0057 (11)	0.0034 (12)
C9	0.0319 (16)	0.0365 (17)	0.0245 (15)	−0.0100 (13)	0.0062 (13)	0.0052 (13)
C10	0.0193 (13)	0.0249 (14)	0.0229 (14)	0.0010 (11)	0.0052 (11)	0.0009 (11)
C11	0.0207 (13)	0.0282 (15)	0.0207 (14)	0.0047 (11)	0.0029 (11)	0.0029 (11)
C12	0.056 (2)	0.0400 (18)	0.0198 (15)	0.0021 (16)	0.0129 (14)	−0.0015 (13)
C13	0.072 (3)	0.106 (4)	0.042 (2)	0.003 (3)	0.013 (2)	0.047 (2)
C14	0.0433 (19)	0.0330 (18)	0.049 (2)	0.0118 (15)	0.0124 (16)	−0.0060 (15)
C7'	0.055 (2)	0.043 (2)	0.039 (2)	−0.0142 (17)	0.0121 (17)	−0.0122 (16)
C9'	0.051 (2)	0.088 (3)	0.052 (3)	0.021 (2)	0.001 (2)	−0.006 (2)
C8'	0.108 (4)	0.073 (3)	0.050 (3)	−0.021 (3)	0.034 (3)	−0.027 (2)

### Geometric parameters (Å, º)

**Table:**

S1—C5	1.749 (3)	C5'B—C4'B	1.491 (7)
S1—C2	1.756 (3)	C5'B—C7'	1.555 (8)
N1—C1'	1.286 (4)	C5'B—H5'B	1.0000
N1—N2	1.405 (3)	C4'B—C3'	1.486 (5)
N2—C2	1.274 (3)	C4'B—H4'3	0.9900
N3—C4	1.393 (3)	C4'B—H4'4	0.9900
N3—C2	1.398 (3)	C3'—C2'	1.330 (4)
N3—C7	1.423 (3)	C3'—H3'	0.9500
O4—C4	1.208 (3)	C2'—C17	1.500 (4)
O11—C11	1.206 (3)	C17—H17A	0.9800
O12—C11	1.331 (3)	C17—H17B	0.9800
O12—C12	1.446 (3)	C17—H17C	0.9800
O61—C6	1.193 (3)	C6—C7	1.508 (4)
O62—C6	1.329 (3)	C7—C8	1.322 (4)
O62—C14	1.441 (4)	C8—C9	1.480 (4)
O91—C9	1.193 (4)	C8—H8	0.9500
O92—C9	1.337 (4)	C10—C11	1.469 (4)
O92—C13	1.447 (4)	C10—H10	0.9500
C4—C5	1.481 (4)	C12—H12A	0.9800
C5—C10	1.331 (4)	C12—H12B	0.9800
C1'—C2'	1.472 (4)	C12—H12C	0.9800
C1'—C6'A	1.492 (14)	C13—H13A	0.9800
C1'—C6'B	1.531 (14)	C13—H13B	0.9800
C6'A—C5'A	1.519 (9)	C13—H13C	0.9800
C6'A—H6'1	0.9900	C14—H14A	0.9800
C6'A—H6'2	0.9900	C14—H14B	0.9800
C5'A—C4'A	1.517 (7)	C14—H14C	0.9800
C5'A—C7'	1.547 (7)	C7'—C8'	1.383 (6)
C5'A—H5'A	1.0000	C7'—C9'	1.425 (6)
C4'A—C3'	1.486 (5)	C9'—H9'1	0.9800
C4'A—H4'1	0.9900	C9'—H9'2	0.9800
C4'A—H4'2	0.9900	C9'—H9'3	0.9800
C6'B—C5'B	1.516 (10)	C8'—H8'1	0.9500
C6'B—H6'3	0.9900	C8'—H8'2	0.9500
C6'B—H6'4	0.9900		
			
C5—S1—C2	90.20 (12)	C2'—C3'—H3'	117.7
C1'—N1—N2	113.9 (2)	C4'A—C3'—H3'	117.7
C2—N2—N1	110.0 (2)	C3'—C2'—C1'	119.1 (3)
C4—N3—C2	114.9 (2)	C3'—C2'—C17	123.1 (3)
C4—N3—C7	123.6 (2)	C1'—C2'—C17	117.8 (3)
C2—N3—C7	121.6 (2)	C2'—C17—H17A	109.5
C11—O12—C12	114.8 (2)	C2'—C17—H17B	109.5
C6—O62—C14	115.3 (2)	H17A—C17—H17B	109.5
C9—O92—C13	115.4 (3)	C2'—C17—H17C	109.5
N2—C2—N3	120.4 (2)	H17A—C17—H17C	109.5
N2—C2—S1	126.7 (2)	H17B—C17—H17C	109.5
N3—C2—S1	112.80 (18)	O61—C6—O62	125.7 (3)
O4—C4—N3	125.0 (2)	O61—C6—C7	123.8 (3)
O4—C4—C5	125.3 (2)	O62—C6—C7	110.5 (2)
N3—C4—C5	109.7 (2)	C8—C7—N3	119.4 (2)
C10—C5—C4	120.1 (2)	C8—C7—C6	124.1 (2)
C10—C5—S1	127.5 (2)	N3—C7—C6	116.4 (2)
C4—C5—S1	112.35 (19)	C7—C8—C9	124.6 (3)
N1—C1'—C2'	116.8 (3)	C7—C8—H8	117.7
N1—C1'—C6'A	123.9 (5)	C9—C8—H8	117.7
C2'—C1'—C6'A	118.5 (5)	O91—C9—O92	124.3 (3)
N1—C1'—C6'B	124.6 (4)	O91—C9—C8	126.6 (3)
C2'—C1'—C6'B	117.4 (5)	O92—C9—C8	108.9 (3)
C1'—C6'A—C5'A	111.6 (8)	C5—C10—C11	121.3 (2)
C1'—C6'A—H6'1	109.3	C5—C10—H10	119.3
C5'A—C6'A—H6'1	109.3	C11—C10—H10	119.3
C1'—C6'A—H6'2	109.3	O11—C11—O12	124.7 (3)
C5'A—C6'A—H6'2	109.3	O11—C11—C10	123.8 (3)
H6'1—C6'A—H6'2	108.0	O12—C11—C10	111.4 (2)
C4'A—C5'A—C6'A	110.3 (6)	O12—C12—H12A	109.5
C4'A—C5'A—C7'	111.9 (4)	O12—C12—H12B	109.5
C6'A—C5'A—C7'	110.5 (7)	H12A—C12—H12B	109.5
C4'A—C5'A—H5'A	108.0	O12—C12—H12C	109.5
C6'A—C5'A—H5'A	108.0	H12A—C12—H12C	109.5
C7'—C5'A—H5'A	108.0	H12B—C12—H12C	109.5
C3'—C4'A—C5'A	113.1 (3)	O92—C13—H13A	109.5
C3'—C4'A—H4'1	109.0	O92—C13—H13B	109.5
C5'A—C4'A—H4'1	109.0	H13A—C13—H13B	109.5
C3'—C4'A—H4'2	109.0	O92—C13—H13C	109.5
C5'A—C4'A—H4'2	109.0	H13A—C13—H13C	109.5
H4'1—C4'A—H4'2	107.8	H13B—C13—H13C	109.5
C5'B—C6'B—C1'	111.8 (8)	O62—C14—H14A	109.5
C5'B—C6'B—H6'3	109.3	O62—C14—H14B	109.5
C1'—C6'B—H6'3	109.3	H14A—C14—H14B	109.5
C5'B—C6'B—H6'4	109.3	O62—C14—H14C	109.5
C1'—C6'B—H6'4	109.3	H14A—C14—H14C	109.5
H6'3—C6'B—H6'4	107.9	H14B—C14—H14C	109.5
C4'B—C5'B—C6'B	111.7 (7)	C8'—C7'—C9'	120.9 (4)
C4'B—C5'B—C7'	112.8 (5)	C8'—C7'—C5'A	127.0 (4)
C6'B—C5'B—C7'	106.7 (7)	C9'—C7'—C5'A	110.3 (4)
C4'B—C5'B—H5'B	108.5	C8'—C7'—C5'B	111.9 (4)
C6'B—C5'B—H5'B	108.5	C9'—C7'—C5'B	125.9 (4)
C7'—C5'B—H5'B	108.5	C7'—C9'—H9'1	109.5
C3'—C4'B—C5'B	115.7 (4)	C7'—C9'—H9'2	109.5
C3'—C4'B—H4'3	108.4	H9'1—C9'—H9'2	109.5
C5'B—C4'B—H4'3	108.4	C7'—C9'—H9'3	109.5
C3'—C4'B—H4'4	108.4	H9'1—C9'—H9'3	109.5
C5'B—C4'B—H4'4	108.4	H9'2—C9'—H9'3	109.5
H4'3—C4'B—H4'4	107.4	C7'—C8'—H8'1	120.0
C2'—C3'—C4'A	124.7 (3)	C7'—C8'—H8'2	120.0
C2'—C3'—C4'B	124.7 (3)	H8'1—C8'—H8'2	120.0

### Hydrogen-bond geometry (Å, º)

**Table:**

*D*—H···*A*	*D*—H	H···*A*	*D*···*A*	*D*—H···*A*
C6′*A*—H6′2···O12^i^	0.99	2.56	3.349 (8)	136
C3′—H3′···O4^ii^	0.95	2.57	3.510 (3)	170
C4′*B*—H4′4···O11^iii^	0.99	2.45	3.414 (4)	164
C10—H10···O62^iv^	0.95	2.47	3.244 (3)	138

Symmetry codes: (i) −*x*+1, −*y*+2, −*z*+1; (ii) *x*−1, *y*−1, *z*; (iii) −*x*+1, −*y*+1, −*z*+1; (iv) −*x*+2, −*y*+2, −*z*+1.

## References

[bb1] Bruker (2014). *APEX3*, *SAINT* and *SADABS*. Bruker AXS Inc., Madison, Wisconsin, USA.

[bb2] Burnett, M. N. & Johnson, C. K. (1996). *ORTEPIII*. Report ORNL-6895. Oak Ridge National Laboratory, Tennessee, USA.

[bb3] Farrugia, L. J. (2012). *J. Appl. Cryst.* **45**, 849–854.

[bb4] Gautam, D. & Chaudhary, R. P. (2015). *J. Mol. Struct.* **1080**, 137–144.

[bb5] Gautam, D., Gautam, P. & Chaudhary, R. P. (2013). *Heterocycl. Commun.* **19**, 43–47.

[bb6] Groom, C. R., Bruno, I. J., Lightfoot, M. P. & Ward, S. C. (2016). *Acta Cryst.* B**72**, 171–179. 10.1107/S2052520616003954 PMC4822653 27048719

[bb7] Gupta, R. & Chaudhary, R. P. (2013). *Phosphorus Sulfur Silicon*, **188**, 1296–1304.

[bb8] Hirai, K. & Sugimoto, H. (1977). *Chem. Pharm. Bull.* **25**, 2292–2299.

[bb9] Le Flohic, A., Meyer, C. & Cossy, J. (2005). *Org. Lett.* **7**, 339–342. 10.1021/ol047603q 15646992

[bb10] Macrae, C. F., Bruno, I. J., Chisholm, J. A., Edgington, P. R., McCabe, P., Pidcock, E., Rodriguez-Monge, L., Taylor, R., van de Streek, J. & Wood, P. A. (2008). *J. Appl. Cryst.* **41**, 466–470.

[bb11] Mague, J. T., Akkurt, M., Mohamed, S. K., Hassan, A. A. & Albayati, M. R. (2014). *Acta Cryst.* E**70**, o436–o437. 10.1107/S1600536814005285 PMC3998598 24826142

[bb12] Mohamed, S. K., Mague, J. T., Akkurt, M. & Albayati, M. R. (2015). Private communication (refcode MUDRIO). CCDC, Cambridge, England.

[bb13] Nielsen, D. S., Hoang, H. N., Lohman, R., Diness, F. & Fairlie, D. P. (2012). *Org. Lett.* **14**, 5720–5723. 10.1021/ol3027347 23130644

[bb15] Ramachandran, R., Rani, M. & Kabilan, S. (2009). *Acta Cryst.* E**65**, o584. 10.1107/S1600536809005339 PMC2968662 21582239

[bb20] Sheldrick, G. M. (2008). *Acta Cryst* A**64**, 112–122. 10.1107/S0108767307043930 18156677

[bb16] Sheldrick, G. M. (2015*a*). *Acta Cryst.* A**71**, 3–8.

[bb17] Sheldrick, G. M. (2015*b*). *Acta Cryst.* C**71**, 3–8.

[bb18] Spek, A. L. (2009). *Acta Cryst.* D**65**, 148–155. 10.1107/S090744490804362X PMC2631630 19171970

[bb19] Uchikawa, O., Fukatsu, K., Suno, M., Aono, T. & Doi, T. (1996). *Chem. Pharm. Bull.* **44**, 2070–2077. 10.1248/cpb.44.2070 8945772

